# Efficient transmission of human prion diseases to a glycan-free prion protein-expressing host

**DOI:** 10.1093/brain/awad399

**Published:** 2023-11-24

**Authors:** Laura Cracco, Ignazio Cali, Mark L Cohen, Rabail Aslam, Silvio Notari, Qingzhong Kong, Kathy L Newell, Bernardino Ghetti, Brian S Appleby, Pierluigi Gambetti

**Affiliations:** Department of Pathology and Laboratory Medicine, Indiana University, School of Medicine, Indianapolis, IN 46202, USA; Department of Pathology, Case Western Reserve University, School of Medicine, Cleveland, OH 44106, USA; National Prion Disease Pathology Surveillance Center, Case Western Reserve University, School of Medicine, Cleveland, OH 44106, USA; Department of Pathology, Case Western Reserve University, School of Medicine, Cleveland, OH 44106, USA; Department of Pathology, Case Western Reserve University, School of Medicine, Cleveland, OH 44106, USA; Department of Pathology, Case Western Reserve University, School of Medicine, Cleveland, OH 44106, USA; Department of Pathology, Case Western Reserve University, School of Medicine, Cleveland, OH 44106, USA; National Prion Disease Pathology Surveillance Center, Case Western Reserve University, School of Medicine, Cleveland, OH 44106, USA; Department of Neurology, Case Western Reserve University, School of Medicine, Cleveland, OH 44106, USA; Department of Pathology and Laboratory Medicine, Indiana University, School of Medicine, Indianapolis, IN 46202, USA; Department of Pathology and Laboratory Medicine, Indiana University, School of Medicine, Indianapolis, IN 46202, USA; Department of Pathology, Case Western Reserve University, School of Medicine, Cleveland, OH 44106, USA; National Prion Disease Pathology Surveillance Center, Case Western Reserve University, School of Medicine, Cleveland, OH 44106, USA; Department of Neurology, Case Western Reserve University, School of Medicine, Cleveland, OH 44106, USA; Department of Psychiatry, Case Western Reserve University, Cleveland, OH 44106, USA; Department of Pathology, Case Western Reserve University, School of Medicine, Cleveland, OH 44106, USA

**Keywords:** glycosylation, transgenic, conformation, histopathology, prion stability, toxic prion

## Abstract

It is increasingly evident that the association of glycans with the prion protein (PrP), a major post-translational modification, significantly impacts the pathogenesis of prion diseases. A recent bioassay study has provided evidence that the presence of PrP glycans decreases spongiform degeneration and disease-related PrP (PrP^D^) deposition in a murine model. We challenged (*PRNP*^N181Q/197Q^) transgenic (Tg) mice expressing glycan-free human PrP (TgGlyc−), with isolates from sporadic Creutzfeldt–Jakob disease subtype MM2 (sCJDMM2), sporadic fatal insomnia and familial fatal insomnia, three human prion diseases that are distinct but share histotypic and PrP^D^ features. TgGlyc− mice accurately replicated the basic histotypic features associated with the three diseases but the transmission was characterized by high attack rates, shortened incubation periods and a greatly increased severity of the histopathology, including the presence of up to 40 times higher quantities of PrP^D^ that formed prominent deposits. Although the engineered protease-resistant PrP^D^ shared at least some features of the secondary structure and the presence of the anchorless PrP^D^ variant with the wild-type PrP^D^, it exhibited different density gradient profiles of the PrP^D^ aggregates and a higher stability index. The severity of the histopathological features including PrP deposition appeared to be related to the incubation period duration. These findings are clearly consistent with the protective role of the PrP glycans but also emphasize the complexity of the conformational changes that impact PrP^D^ following glycan knockout. Future studies will determine whether these features apply broadly to other human prion diseases or are PrP^D^-type dependent.

## Teaser

Cracco *et al.* study the transmission of three human prion diseases to a double mutant transgenic mouse expressing a glycan-free human prion protein, and show the protective role of prion protein glycans. The transgenic mouse model represents a unique tool to resolve the structure of human prion strains with atomic resolution.

## Introduction

Two major post-translational modifications are associated with the normal or cellular prion protein (PrP^C^): N-linked glycosylation and the addition of the glycophosphatidylinositol (GPI) anchor.^1^ The glycosylation is non-obligatory and in human PrP^C^, it occurs at the two consensus sites Asn (N) 181 and N197.^2,3^ These features result in the coexistence of four PrP^C^ glycoforms that distribute over three electrophoretic bands: diglycosylated, monoglycosylated at either N181 or N197 residue, and unglycosylated.^4^

After years of controversy, the impact of glycosylation on the templated conversion of PrP^C^ to the abnormal and disease-related PrP (PrP^D^) has recently been clarified.^2,5-14^ Most of the controversy focused on the mutational approach used to generate the experimental models expressing glycan-free PrP. Systematic studies in cell and, more recently, transgenic (Tg) mouse models have shown that the Asn to Gln (N-to-Q) mutation of the PrP^C^ gene at both consensus sites is the least disruptive for unglycosylated PrP expression, cell trafficking and final cell destination. In contrast, the substitution used in previous studies resulted in the retention of the glycan-free PrP in intracellular compartments rather than becoming plasma membrane-bound like wild-type (WT) PrP^C^.^2,3,7,8,15-17^ Recent bioassays in a knock-in mouse model expressing glycan-free PrP following the N-to-Q mutation at both glycosylation sites showed that glycans significantly impact prion strain formation and the histopathological phenotype or histotype.^3,17^ However, these remarkable studies were conducted in murine models, which limits their translational value to human diseases.

The human prion diseases exhibit a unique range of clinical and histopathological phenotypes, along with their associated PrP^D^ strains that may vary in major features, such as the size of the proteinase K-resistant (res) core and the relative representation of the glycan components.^4,18-21^ Furthermore, human prions display distinct competencies to self-replicate upon bioassay, resulting in varying attack rates, incubation periods and disease phenotypes.^22^

This heterogeneity is rooted in several features that are especially relevant to human prion diseases. These include (i) three aetiologies (idiopathic, inherited and acquired); (ii) the disease-modifying common methionine (M)/valine (V) polymorphism at codon 129 of the PrP gene (*PRNP*), which generates distinct PrP allotypes; and (iii) the presence of two major variants of PrP^D^ known as type 1 and 2, along with several other minor variants that are mostly distinguished by the size of their protease-resistant cores as well as type and distribution of the histopathological lesions or histotype.^4,20,23^ Human prion diseases include familial and sporadic forms of Creutzfeldt-Jakob disease (f- and sCJD) with their subtypes, sporadic and familial fatal insomnia (sFI and FFI), the sporadic variable protease-sensitive prionopathy and the familial prion diseases referred to as prion protein amyloidosis.^24-28^

We engineered a homozygous Tg mouse that carries the N-to-Q double mutation at codons 181 and 197 of the human PrP gene coupled with methionine at position 129, which here is referred to as TgGlyc−.^15,29^ In an earlier *in vitro* study, we observed the efficiency of the TgGlyc− mice PrP in converting to PrP^D^.^15^ We then challenged TgGlyc− and previously developed control mice (TgGlyc+)^30,31^ with brain homogenates from major subtypes of sporadic and familial human prion diseases.

We now report on the impact that PrP glycosylation knockout has on the histotype of mice and the characteristics of mouse-adapted human PrP^D^ following transmission to TgGlyc− mice and controls of sCJDMM2, sFI and FFI, three distinct diseases that share the 129MM allotype and PrP^D^ type 2.^4,19,27^ Preliminary results of this study have been presented.^32,33^

## Materials and methods

### Transgenic mice

The TgGlyc− mice, commonly identified as TgNN6, have been described previously.^15,29^ They express human PrP lacking both glycans in a 129M background due to the N181Q/N197Q double mutation. Following breeding to homozygosity, their approximate expression level is 0.6 that of the wild-type FVB mice. The TgGlyc+ mice used as controls, originally described as Tg40h, express human PrP^C^ at about twice the normal mouse brain level.^30,31^

### Inocula

Inocula were prepared from the cerebral cortex of three definitively diagnosed cases of sCJDMM2, two cases of sFI and one case of FFI (9–12 months disease duration). All samples were obtained from the National Prion Disease Pathology Surveillance Center (NPDPSC).

### Ethics approval and consent to participate

All procedures were performed under protocols approved by the Institutional Review Board at Case Western Reserve University. Written informed consent for research was obtained from all patients or legal guardians according to the Declaration of Helsinki. All patients’ data and samples were coded and handled in accordance with NIH guidelines to protect patients’ identities.

### Intracerebral inoculation

Intracerebral inoculation to the Tg mice was carried out with 1% homogenate (w/vol) prepared in PBS, as previously described.^30,31^

### Histology and lesion profiles

Histological and immunohistochemical examinations were carried out at four major brain levels at approximately bregma 0.5 mm, −1.7 mm, −3.8 mm and −6.0 mm and stained with haematoxylin and eosin and immunohistochemically, as previously described, using the antibody (Ab) 3F4 to human PrP (residues 106–110)^31,34^; selected sections were processed for immunostaining with antibodies to glial fibrillary acidic protein (GFAP) and microglia (Iba1). Lesion profiles were assessed according to minor modifications of a previously described procedure^35^ using the semiquantitative evaluation of spongiform degeneration severity rated on a 0–5 scale on haematoxylin and eosin-stained sections (0 = not detectable; 1 = minimal, 2–5 = moderate to extremely severe according to the rough estimate of the brain surface occupied by spongiform degeneration). Mouse brain anatomy and nomenclature were applied according to Allen Reference Atlas—Mouse Brain (brain atlas, available from atlas.brain-map.org).

### Electrophoresis and immunoblots

Procedures were performed as previously described in detail.^19^ Densitometric analysis was performed using the Odyssey application software V3.0 (LI-COR Biosciences). After normalization, the data were plotted using GraphPad Prism 9 and expressed as mean ± standard deviation (SD) unless otherwise indicated. Densitometric analysis of the 17 and 19 kDa western blot bands in TgGlyc− mice was performed as described by Notari and colleagues.^36^

### Proteinase K digestion

Samples in 1× lysis buffer (LB) 100 pH 8.0 obtained mixing 20% brain homogenates (w/vol) with 2× LB pH 8.0 were incubated at 37°C for 1 h with either 5 U/ml or 10 U/ml of proteinase K (PK, 58 U/mg specific activity, 1 U/ml equal to 17.2 μg/ml PK). The reaction was stopped by the addition of 3 mM phenylmethanesulphonyl fluoride (PMSF) and proteins were then precipitated in methanol.^19^

### Conformational stability and solubility assay

The conformational stability and solubility assay (CSSA) was performed as originally described, with minor modifications.^19,35,37^

### Sedimentation equilibrium

Sedimentation equilibrium was performed according to Cracco *et al*.^19^

### Study approval

Animal studies were performed under the protocols approved by CWRU Institutional Animal Care and Use Committee (IACUC).

### Reagents and antibodies

Sodium dodecyl sulphate (SDS), β-mercaptoethanol, Tween 20, 15% Criterion Tris-HCl polyacrylamide precast gels, bromophenol blue, non-fat dry milk, Tris-buffered saline (TBS) were purchased from Bio-Rad Laboratories. NaCl, Nonidet P-40, sodium deoxycholate, Tris-HCl, PBS, Dulbecco’s PBS (D-PBS), *N*-Lauroylsarcosine sodium salt solution (sarkosyl NL), PMSF, PK, sucrose, Kodak Biomax MR and XAR films were ordered from MilliporeSigma; Odyssey Blocking Buffer from LI-COR Biosciences; methanol, GdnHCl solution from Thermo Fisher Scientific Inc.; EDTA from Promega; polyvinylidene difluoride (PVDF) membrane (Immobilon-P or Immobilon-FL) from EMD Millipore and ECL and ECL plus reagents from GE Healthcare Life Sciences. Antibodies used were primary mouse mAb 3F4 (to human PrP residues 106–110), Tohoku-2 (to human PrP residues 97–103),^38^ GFAP Ab and Microglia Ab Iba1 (Biocare Medical), secondary antibodies IRDye 680RD goat anti-rabbit IgG (LI-COR Biosciences) and sheep anti-mouse IgG, HRP-linked whole antibodies from GE Healthcare, Life Sciences.

### Statistics

Statistical analysis was performed using GraphPad Prism 9 and Excel software. Unpaired two-tailed Student’s *t*-test or Welch’s *t*-test were used after determining equal or unequal variance between the samples. *P* ≤ 0.05 was considered statistically significant.

## Results

### Transmission features following inoculation of sCJDMM2, sporadic and familial fatal insomnia homogenates to TgGlyc+ and TgGlyc− mice

Transmission features are summarized in Table 1 and described below.

**Table 1:** Inocula, attack rates, incubation periods and templated PrP^D^ types following transmission of sCJDMM2, sporadic and familial fatal insomnia to TgGlyc+ and TgGlyc− mice

TgGlyc+			TgGlyc−		
Inoculum	Attack rate	Incubation period (dpi)^a^	Inoculum	Attack rate	Incubation period (dpi)^a^
**sCJDMM2**			**sCJDMM2**		
First passage A^b^	5/6	557 ± 56	First passage A, B and C^b^	6/6; 9/10; 8/8	279 ± 5; 250 ± 70; 274 ± 12
First passage B^b^	5/7	582 ± 142	Second passage	8/8	78 ± 0
—	T2^c^: 3/7	695 ± 81	Third passage	10/10	77 ± 8
—	T1^c^: 4/7	503 ± 62			
**Sporadic fatal insomnia**			**Sporadic fatal insomnia**		
First passage	5/5	612 ± 40	First passage A and B^b^	7/8; 4/7	260 ± 20; 497 ± 49
			Second passage A and B^b^	7/7; 7/7	77 ± 10; 227 ± 9
			Third passage	11/11	76 ± 5
**Familial fatal insomnia**			**Familial fatal insomnia**		
First passage	0/11	791 ± 31^e^	First passage	3/10	289 ± 13
TgGlyc− adapted^d^	0/8	667 ± 87^e^	Second passage	8/8; 7/7	79 ± 11; 306 ± 19
			**Drowsy and hyperactive**	0/4 and 0/8	720 ± 144^e^; 676 ± 123^e^
			**None**	0/6	713 ± 80^e^; 618–832^f^

sCJDMM2 = sporadic Creutzfeldt–Jakob disease subtype MM2.

The incubation period is expressed as mean days post-inoculation (dpi) ± standard deviation (SD).

^b^The passages indicated as A, B or C throughout the table were carried out inoculating homogenates from distinct human cases at first passage or from distinct positive transgenic (Tg) mice at second and third passages.

^c^T (type) 1 and 2 of disease-related prion protein (PrP^D^) is based on western blot examinations.

^d^Inoculum from a positive familial fatal insomnia-inoculated TgGlyc− mouse.

^e^Lifespan average ± SD.

^f^Lifespan range of uninoculated TgGlyc− mice.

#### TgGlyc+ mice

For sCJDMM2, two transmission experiments using brain homogenate from two donors showed attack rates ranging from 70% to 80% with similar incubation periods averaging 570 days post-inoculation (dpi). The resulting resPrP^D^ was either type 1 or type 2 and these two types were consistently replicated in different mice. Although the attack rates of the mice from the two resPrP^D^ type groups were similar at ∼50%, the incubation period was significantly longer in the mice associated with type 2 than type 1 (695 ± 81 versus 503 ± 62 dpi; *P* = 0.016).

Sporadic FI showed a 100% attack rate with an incubation period of 612 ±40 dpi, while direct transmission of FFI failed. Remarkably, FFI did not transmit to TgGlyc+ even after a previous positive passage through TgGlyc− mice.

#### TgGlyc− mice

Following inoculation of brain isolates from three sCJDMM2 donors, the first passage attack rates were almost 100%, with comparable incubation periods averaging 266 ± 44 dpi (Table 1). At the second and third passages, attack rates were invariably 100% with incubation periods reduced to 77 and 78 dpi. In contrast, sFI showed attack rates of 90% and 60% in the two first passage experiments, with incubation periods of 260 ± 20 dpi and 497 ± 49 dpi, respectively. The attack rates increased to 100% in the two second passage transmissions, with variable incubation periods of 77 ± 10 and 227 ± 9 dpi, respectively. The FFI attack rate at first passage was 30%, the lowest of the three conditions, while the incubation period of 289 dpi was consistent with those of the previous two conditions (Table 1). The second passage experiments that utilized two first passage FFI-inoculated TgGlyc− mice, shared a 100% attack rate, but the incubation periods varied (79 ± 11 versus 306 ± 19 dpi). Furthermore, the transmission of the hamster ‘Drowsy’ and ‘Hyperactive’ prion strains to test the existence of the species barrier in the TgGlyc− mice failed.^39,40^ Finally, uninoculated TgGlyc− mice lived for over 800 days without any detectable histopathology, making spontaneous prion disease in these mice unlikely (Table 1).

### Histopathology

#### Human sCJDMM2

**Figure 1:**
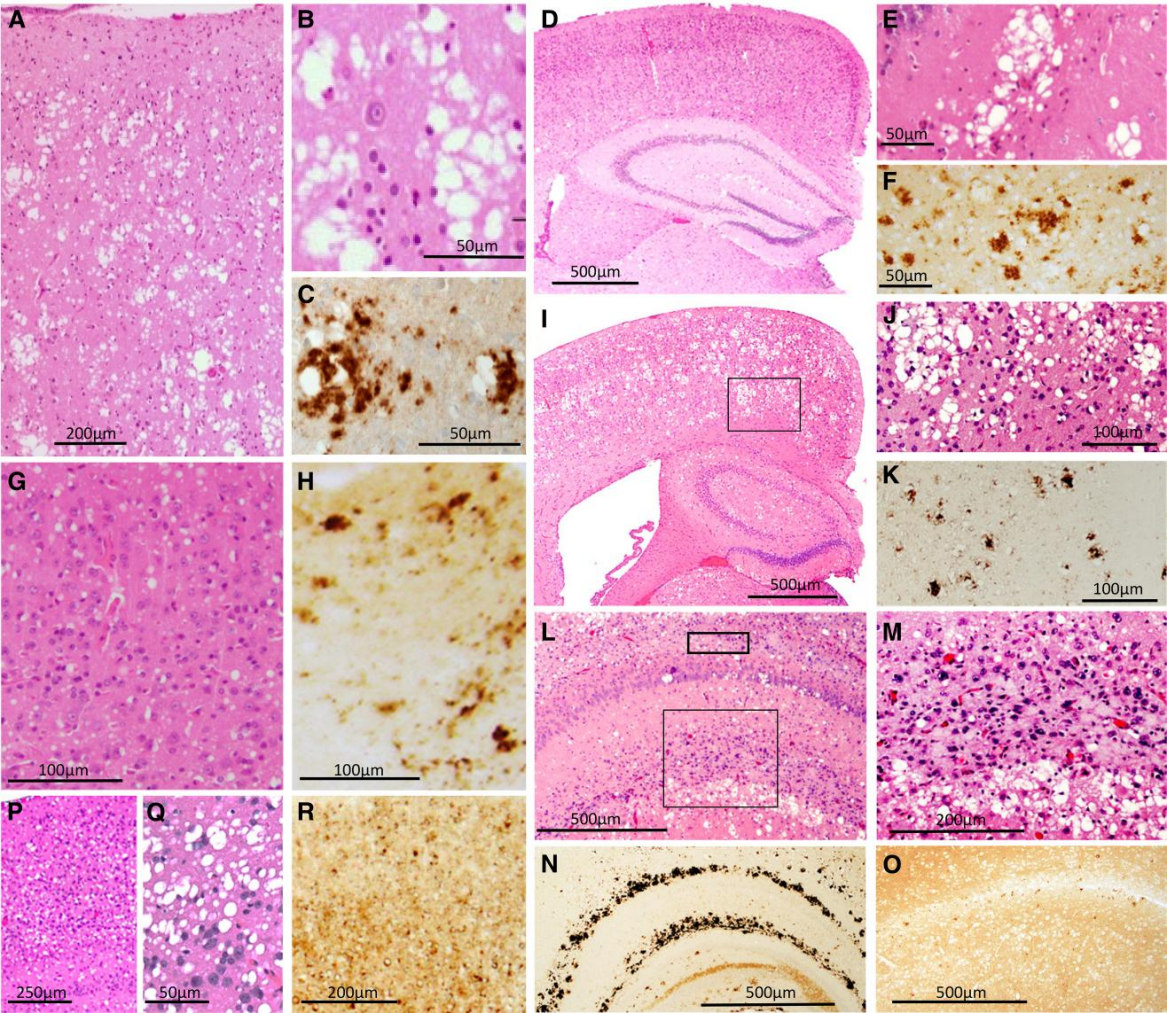
**Histopathology of the human sCJDMM2 and of TgGlyc+ and TgGlyc− mice following inoculation with sCJDMM2 isolates.** (**A**–**C**) sCJDMM2 spongiform degeneration predominantly impacting the cerebral cortex is typified by the presence of large vacuoles that are often clustered, confluent (**A** and **B**), and associated with PrP^D^ deposits (**C**). (**D**–**F**) In sCJDMM2-challenged TgGlyc+ mice replicating PrP^D^ type 2 (incubation average 695 dpi) the spongiform degeneration type (**E**, from hippocampus) and PrP^D^ immunostaining pattern (**F**) mimicked those of sCJDMM2 but these changes were rare and scattered (**D**). (**G** and **H**) In sCJDMM2-challenged TgGlyc+ mice templating type 1, spongiform degeneration showed small non-clustered vacuoles resembling those of the sCJDMM1 subtype (**G**) while PrP^D^ formed (**H**) a mixture of relatively large and punctate aggregates. (**I**–**N**) TgGlyc− mice at first passage (incubation 279 dpi) accurately reproduced the original spongiform degeneration type and the PrP^D^ deposition pattern but with significantly greater severity (**I** and **J**) (**J** is the enlargement of the cortical region framed in **I**) (see also Fig. 2A). (**L**–**N**) Two elongated PrP^D^ deposits, the larger of which was observed in the hippocampal stratum lacunosum-moleculare and the smaller in the outer parahippocampal regions, are indicated by the large and small frames, respectively, in **L**. (**M**) Higher magnification of the larger area framed in **L**, shows mildly basophilic deposits consistent with amyloid, but without detectable core, mixed with an intense cellular reaction (see also Fig. 2). (**N** and **O**) Following PrP immunostaining, the basophilic deposits looked like a curved railroad track that was not observed in the TgGlyc+ (**O**). At third passage (incubation 76 dpi, **P**–**R**), the vacuoles were large but they appeared to be less clustered and mostly non-confluent with finer PrP^D^ deposition. Haematoxylin and eosin and antibody 3F4 to PrP were used in Figs 1, 3 and 4. dpi = days post inoculation.

The histotype is characterized by a morphologically distinct spongiform degeneration associated with large and often confluent vacuoles, whose rims often immunostain intensely with PrP antibodies (Fig. 1A–C). The spongiform degeneration impacts to a greater degree the cerebral cortex but it is rare in the cerebellum and brainstem. Although sCJDMM2 is typically associated with PrP^D^ type 2,^27^ both PrP^D^ types may co-occur with variable ratios in a significant number of cases.^41^

#### sCJDMM2 transmission to TgGlyc+ mice templating PrP^D^ type 2: incubation 695 dpi

**Figure 2:**
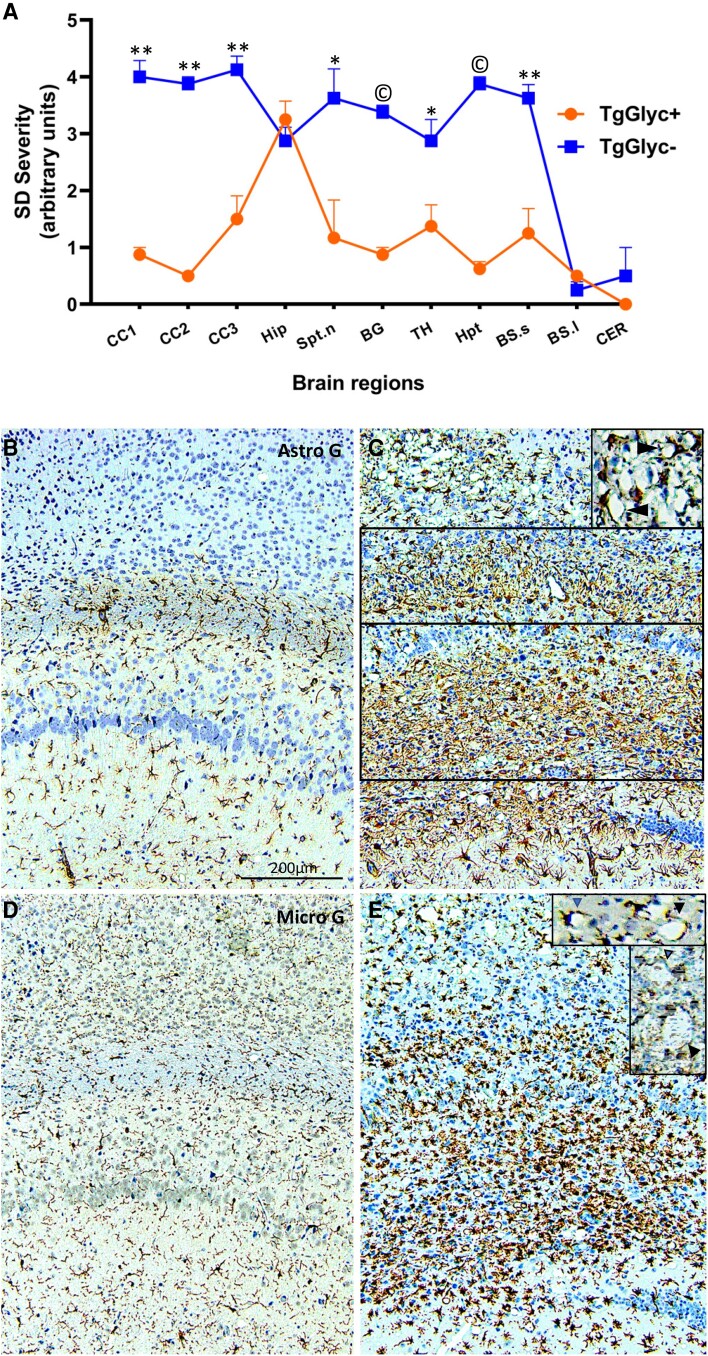
**Spongiform degeneration profile and glial reaction in TgGlyc− and TgGlyc+ mice following inoculation of sCJDMM2 isolates.** (**A**) The profile of the spongiform degeneration (SD) impacting the TgGlyc− mice (incubation average 266 dpi) was significantly more severe than that of the TgGlyc+ controls (first passage, 695 dpi) in all the cerebral grey structures examined except for the hippocampal formation (Hip) where the spongiform degeneration scores overlapped. Lower brainstem and cerebellum were minimally impacted in both TgGlyc mouse groups. *P < 0.05; ** to ^©^*P* < 10^−2^ to <10^−5^; vertical bars at profile values refer to ± standard error of the mean (SEM). CC1–3 = hemispheric cerebral cortex superior, middle, inferior regions; Hip = hippocampus; Spt. n = septal nuclei; BG = basal ganglia; TH = thalamus; Hpt = hypothalamus; BS.s and l = brainstem superior and lower; CER = cerebellum. (**B**–**E**) Both astroglial (Astro) reaction (**B** and **C**) and microglial (Micro) activation (**D** and **E**) were very severe in the two major hippocampal PrP^D^ deposits noticeable in TgGlyc− mice (**C**, **E** and framed in **C**) but not in the controls (**B** and **D**); both glial responses also co-distributed with the spongiform degeneration (**C** and **E**, *top*) and were much stronger in the TgGlyc− mice than in controls. Of note, both reactive astrocytes and activated microglia were interspersed within the spongiform degeneration and seemed to surround some of the spongiform degeneration vacuoles^42^ (**C** and **E***insets*). Antibodies: GFAP to astrocytes, Iba1 to microglia; all panels share the magnification; *insets* magnification = ×∼2.

The spongiform degeneration reproduced the large vacuoles often clustered and confluent of the human disease, although they were rare (Fig. 1D and E). The spongiform degeneration preferentially populated the hippocampal formation while it remained moderate-to-minimal in all other brain regions sparing the cerebellum (Fig. 2A). There was no detectable loss of hippocampal pyramidal cells or of other neuronal populations, and the astroglia and microglia reactions were minimal (Fig. 2B and D). PrP immunostaining was mostly limited to the clusters of large vacuoles and predominantly comprised coarse granules sometimes merging into larger aggregates without core plaques formation (Fig. 1F).

#### sCJDMM2 transmission templating PrP^D^ type 1: incubation 503 dpi

The transmission was characterized by widespread spongiform degeneration predominantly made of small-size vacuoles mimicking those associated with sCJDMM1 (Fig. 1G) while the PrP immunostaining pattern was often punctate and mixed with occasional larger aggregates (Fig. 1H).

#### sCJDMM2 transmission to TgGlyc− mice, first passage: three experiments with average incubation of 266 dpi

The spongiform degeneration with the characteristic large vacuoles mimicked that observed in TgGlyc+ mouse brains associated with PrP^D^ type 2, but it was more severe and widespread impacting the cerebral cortex, often spanning the entire cortical thickness as well as subcortical formations (Fig. 2A). The spongiform degeneration also showed the characteristic PrP immunostaining pattern (Fig. 1I–K) and was accompanied by a cellular reaction (Fig. 2C and E). The C1 region of the hippocampal pyramidal cell layer was significantly depopulated, and, in the outer hippocampus region, brain tissue was replaced by two elongated and aligned basophilic deposits also associated with prominent cellular infiltration (Figs 1L and M and 2C and E). The PrP immunostaining of the two deposits formed a railroad track-like image of immunostained granules, which was not observed in the TgGlyc+ mice (Fig. 1N and O). Smaller basophilic deposits were also seen in the septal nuclei and focally in the neocortex. The drastic difference in severity of the histopathological lesions between TgGlyc− and TgGlyc+ mice at first passage was further supported by the spongiform degeneration profiling and the immunohistochemical evaluation of the astrocytic and microglial reactions (Fig. 2). On average the cerebral spongiform degeneration was four times more severe in TgGlyc− mice than in controls except for the hippocampus where the spongiform degeneration severity scores matched (Fig. 2A). Furthermore, the glial response that was minimal in controls, became overwhelming in the TgGlyc− mice especially within and around the PrP^D^ hippocampal deposits but also in spongiform degeneration-impacted regions where glial cells of both types appeared to surround individual vacuoles (Fig. 2B–E).

#### Second and third passages: incubation average 77 dpi

The spongiform degeneration type and distribution mimicked those of the first passage but with reduced severity and fewer vacuole aggregates. PrP immunostaining demonstrated an overall finer granular staining pattern and the lack of large PrP deposits (Fig. 1P–R).

#### Human sporadic fatal insomnia

**Figure 3:**
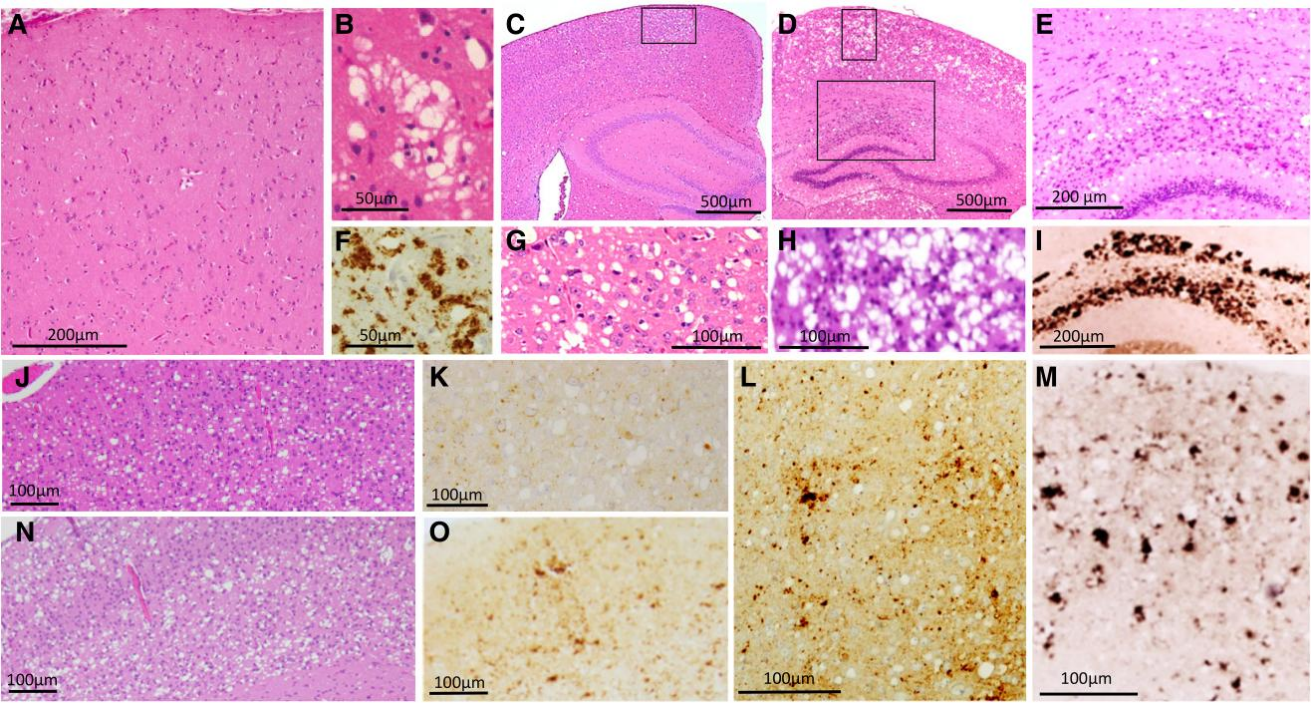
**Histopathology of the human sporadic fatal insomnia and of TgGlyc+ and TgGlyc− mice following inoculation of sporadic fatal insomnia isolates.** Up to 18 months duration (**A**, **B** and **F**) typically there is minimal or no spongiform degeneration in the cerebral cortex (**A**), while beyond 2 year duration, spongiform degeneration and the PrP immunostaining pattern although focal mirror those of sCJDMM2 (**B** and **F**). In addition, sporadic fatal insomnia (sFI) typically presents with severe atrophy of anterior and dorsomedial thalamic nuclei similar to that of familial fatal insomnia (FFI) (see Fig. 4). (**C**, **G** and **L**) In TgGlyc+ mice (612 dpi), the spongiform degeneration was composed of mostly large and discrete vacuoles (**C** and **G**) and PrP^D^ deposits of various sizes (**L**); (**G**: magnified region framed in **C**). TgGlyc− mice at first passage (260 dpi) (**D**, **H** and **M**) were affected by prominent and widespread spongiform degeneration and PrP^D^ deposition that resembled, with a lower degree of severity, the histopathological features of the sCJDMM2-challenged TgGlyc− mice (**D**, **H** and **M**) (see also Fig. 1**J**). These similarities included the large PrP^D^ aggregates of the hippocampus (**E**) forming the railway track-like PrP immunostaining pattern (**I**) (**H**, 90° left rotated and **E** are enlargements of the two regions framed in **D**). (**J**, **K**, **N** and **O**) The histotype of the two second passages with different incubation periods (77 and 227 dpi), showed spongiform degeneration with fewer and apparently smaller vacuoles, associated with lesser PrP^D^ deposition in mice with the shorter incubation (**J** and **K**) compared with those where incubation was longer (**N** and **O**).

Typically, the histotype is characterized by (i) severe neuronal loss with reactive astrogliosis, but no spongiform degeneration, in the anterior and medial dorsal nuclei and the pulvinar (see also the ‘Human familial fatal insomnia’ section)^27^; and (ii) cortical spongiform degeneration, which is directly related to the disease duration as it typically becomes significant when the course exceeds 24 months (Fig. 3A and B).^27^ When it is present, the spongiform degeneration impacts the cerebral cortex by mimicking focally the spongiform degeneration associated with sCJDMM2 for the vacuole characteristics and the immunostaining pattern (Fig. 3F).

#### Sporadic fatal insomnia transmission to TgGlyc+ mice: incubation 612 dpi

The spongiform degeneration was mostly made of large and medium-sized vacuoles with a low tendency to merge while the PrP immunostaining was mostly punctate and with occasional larger PrP deposits corresponding to the rare vacuole clusters (Fig. 3C, G and L). No thalamic atrophy was detected while basal ganglia, cerebellum and brainstem showed minimal or no pathology. Compared to the sCJDMM2-inoculated TgGlyc+ mice, the sFI mice showed a lower proportion of large confluent vacuoles along with overall fewer typical deposits after PrP immunostaining (*cf*. Figs 1E and F and 3G and L).

#### Sporadic fatal insomnia transmission to TgGlyc− mice: first passages with short and long incubation periods

Two first passages with short (260 dpi) and long (497 dpi) incubation periods were examined. Although the lesion severity varied somewhat, the spongiform degeneration was widespread and mimicked the spongiform degeneration features and distribution seen in the matching CJDMM2-inoculated mice but with slightly less severity (Fig. 3D, H and M). Similarly, pyramidal cells were decreased in the hippocampus, which showed PrP-positive basophilic deposits matching those observed in the sCJDMM2-inoculated mice (Fig. 3E and I). Following the 497 dpi incubation, some of the mice showed a similar but even more advanced pathology.

#### Second passage transmission experiments with short and long incubation periods

At the second passage, two transmission experiments with short (77 dpi) and long (227 dpi) incubation periods were seeded with the extracts from the two first passages, respectively (Table 1). The short incubation mice showed a spongiform degeneration associated with medium and large but generally non-confluent vacuoles that mimicked TgGlyc+ mice (*cf*. Fig. 3G and J). The loss of hippocampal pyramidal cells with reactive gliosis and the PrP immunostaining were minimal and there were no large hippocampal PrP deposits (Fig. 3K). Following the 227 dpi incubation, spongiform degeneration resembled more that of the first passage but with diminished vacuole confluency and PrP immunostaining, as well as the lack of hippocampal PrP deposits (Fig. 3N and O).

#### Third passage: incubation 76 dpi

At the third passage, the histotype resembled that of the 77 dpi, second passage.

#### Human familial fatal insomnia: 129MM

**Figure 4:**
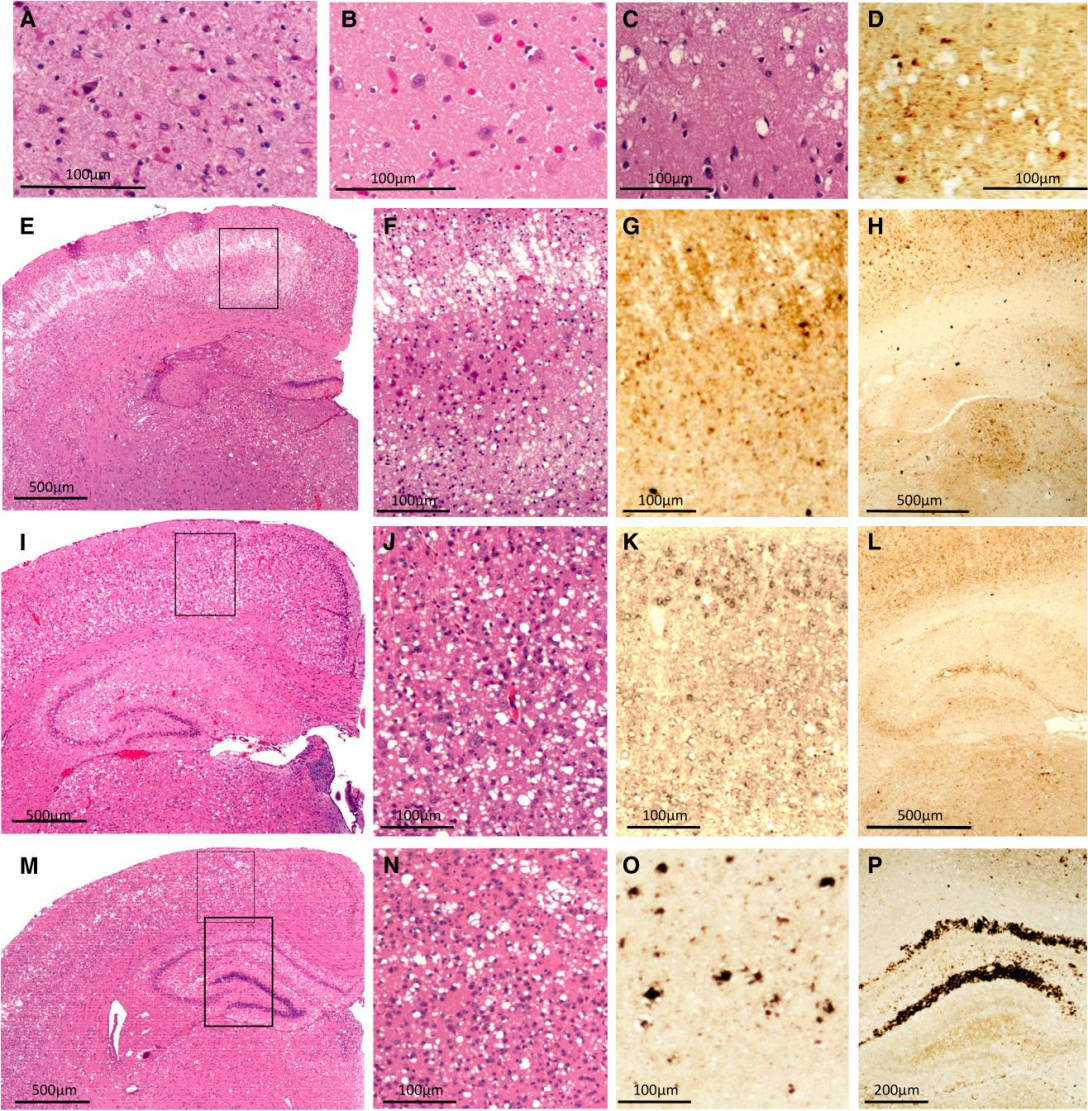
**Histopathology of human familial fatal insomnia and TgGlyc− mice following inoculation of familial fatal insomnia isolates.** The histotype of the familial fatal insomnia (FFI)-129MM subtype (9–12 months duration) (**A**–**D**) mimics that of sporadic fatal insomnia (sFI) including the severe, although focal, thalamic atrophy. (**A**) Intense astrogliosis with neuronal depopulation compared with the normal control (**B**). (**C** and **D**) The spongiform degeneration and PrP^D^ deposition pattern in cases of long duration (>18 months). TgGlyc− mice at first passage (289 dpi) (**E**–**H**) displayed an unusual laminar and occasionally bilaminar spongiform degeneration in the cerebral cortex (**E** and **F**) consisting of adjacent large vacuoles that were vertically aligned (**F**), while isolated vacuoles of various sizes populated the lower cortical region; PrP immunostaining (**G** and **H**) consisted of small PrP^D^ deposits throughout the cerebral cortex (**G**), while the characteristic PrP^D^ aggregates of the hippocampal region were not detected (**H**); (**F** and **G** are enlargements of the cortical area framed in **E**). (**I**–**P**) Second passages with short and long incubation periods (79 and 306 dpi) illustrate the incubation-related difference in histopathological features. Vacuoles were predominantly large but rarely confluent and associated with fine PrP^D^ deposition in the short incubation transmission (**J** and **K**); while vacuolization and PrP^D^ deposition were often typical after the longer incubation, and co-existed with the distinct PrP^D^ deposition in the hippocampus (**M**–**P** and **P** versus **L**). The framed region in **I** and the smaller framed region in **M** are enlarged in **J** and **N**, respectively, while the larger framed area in **M**, is shown after PrP immunostaining, in **P**. PrP = prion protein; PrP^D^ = disease-related prion protein; Tg = transgenic.

The histotype is similar to that of sFI with matching thalamic atrophy, spongiform degeneration features and PrP immunostaining, which are also linked to the disease duration (Fig. 4A–D).^27,43-45^

#### Familial fatal insomnia transmission to TgGlyc+ mice

Transmission failed even with positive inocula previously passaged in TgGlyc− mice.

#### Familial fatal insomnia transmission to TgGlyc− mice first passage: incubation 289 dpi

Spongiform degeneration was made of relatively large vacuoles, whose distribution appeared to be related to their laminar patterns in the cerebral cortex. In the superficial layers, the vacuoles were confluent and formed vertically aligned clusters while in the deeper layers, they were mostly non-confluent (Fig. 4E and F). The PrP immunostaining pattern was mostly characterized by small coarse deposits co-distributed with both patterns of spongiform degeneration while distinct PrP deposition patterns were not detected in the hippocampus (Fig. 4G and H). Thalamic nuclei and hypothalamus were affected by spongiform degeneration but did not show the distinct atrophy of the human disease.

#### Second passages with short and long incubation periods

In the mice with short disease incubation (79 dpi), the spongiform degeneration was predominantly made of large but often non-contiguous vacuoles (Fig. 4I and J). There was moderate and focal loss of pyramidal neurons with reactive gliosis in the hippocampus while the PrP immunostaining mimicked that of the first passage with no major hippocampal PrP deposition (Fig. 4K and L). Following the longer disease incubation (306 dpi) (Fig. 4M–P), the spongiform degeneration vacuoles formed the typical confluent clusters predominantly in the superficial cortical layers (Fig. 4M and N) and an immunostaining pattern similar to that associated with passages requiring long incubation periods in the previous two diseases (Fig. 4O). Similarly, the basophilic PrP deposits with the typical immunostaining pattern were also detected (Fig. 4P).

### Conformational and quantitative studies

#### Western blots and resPrP^D^ quantitative analysis

**Figure 5:**
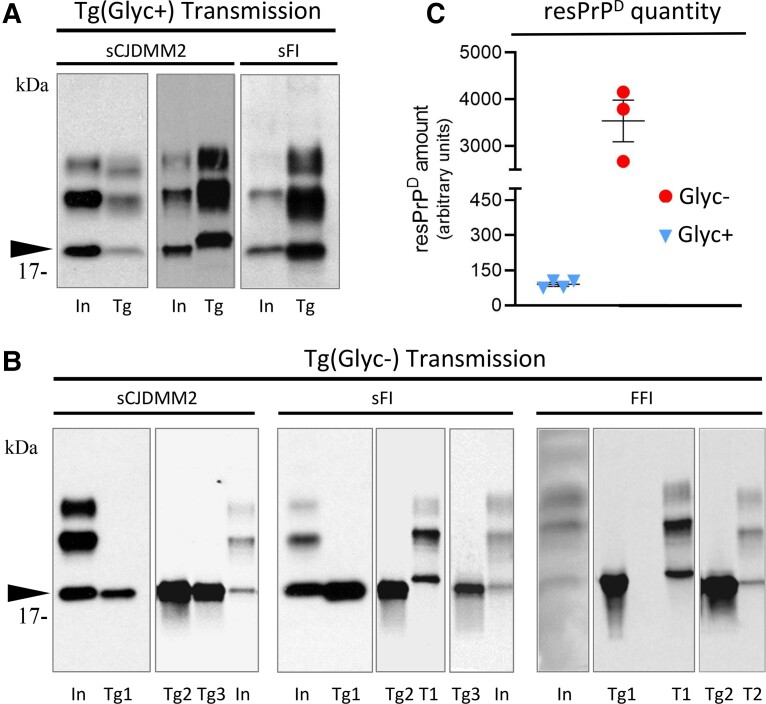
**Western blots with quantitative analysis of resPrP^D^ from inocula and recipient TgGlyc− and TgGlyc+ mice**. (**A** and **B**) For each transmission, lanes identify the respective inoculum (In), or the transgenic donor (Tg). (**B**) The numbers 1–3 identify the passages in transgenic mice; T1 and T2 = resPrP^D^ types 1 and 2 controls. (**A**) In TgGlyc+ mice challenged with sCJDMM2 resPrP^D^ self-replicated either as type 2, like the inoculum (shown in the first panel), or as type 1 (second panel); in contrast, type 2 was always replicated after inoculation with sporadic fatal insomnia (sFI). (**B**) TgGlyc− invariably replicated PrP^D^ type 2. Note the apparently identical electrophoretic mobilities of the TgGlyc− resPrP^D^ and that of the unglycosylated resPrP^D^ conformer of the inoculum that also matches the similar co-mobility in the TgGlyc+ control (arrowhead in **A** and **B**). (**C**) The quantitative analysis of resPrP^D^ recovered from sCJDMM2 inoculated TgGlyc− mice at first passage revealed ∼40× more resPrP^D^ than in controls (3539 versus 90.25; *n* = 4 and *n* = 3 for TgGlyc− and TgGlyc+ mice, first passage, respectively; *P* < 0.02). (**A** and **B**) Ab = 3F4; (**C**) Ab = Tohoku-2. FFI = familial fatal insomnia; resPrP^D^ = resistant disease-related prion protein.

In TgGlyc+ mice both inocula from sCJDMM2 showed the mobilities of either resPrP^D^ type 1 or 2 (always in different mice), while all sFI hosts invariably replicated type 2 (Fig. 5A). Conversely, TgGlyc− mice replicated glycan-resPrP^D^ always type 2 following inoculation of each of the three conditions (Fig. 5B). Of note, the electrophoretic mobility of the resPrP^D^ self-templating in the TgGlyc− mice matched those of the unglycosylated glycoform recovered from the inoculum and the control mice (Fig. 5A and B).

The quantitative analysis revealed that at sCJDMM2 first passage, the resPrP^D^ recovered from the TgGlyc− mice exceeded by nearly 40 times that of the TgGlyc+ mice (90.25 versus 3539; *P* < 0.02) (Fig. 5C).

#### Sedimentation equilibrium

**Figure 6:**
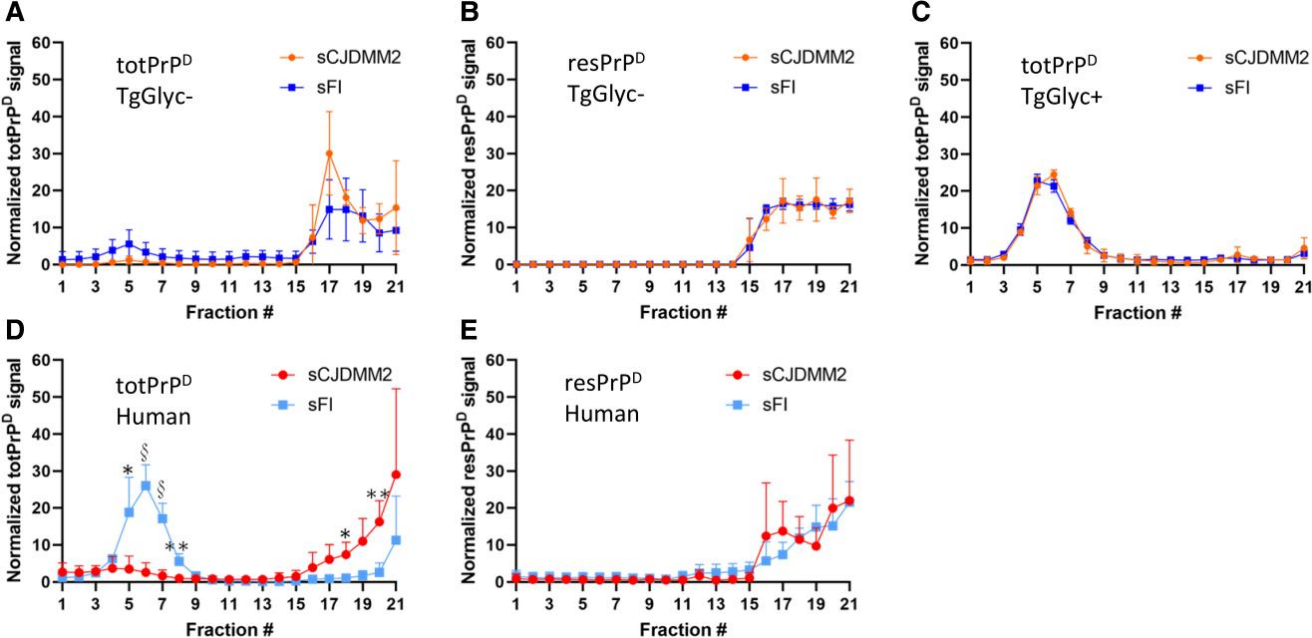
**Sedimentation equilibrium profiles of totPrP^D^ and resPrP^D^ obtained from TgGlyc− and TgGlyc+ mice challenged with sCJDMM2 or sporadic fatal insomnia as well as from isolates of the two human diseases.** (**A** and **D**) Sedimentation equilibrium profiles of totPrP^D^ [proteinase K, PK-resistant (res)PrP^D^ + PK-sensitive PrP^D^] from sCJDMM2- or sporadic fatal insomnia (sFI)-inoculated TgGlyc− mice (**A**) show the near loss of the bell profile encompassing fractions 3 to 9 that distinguish the two diseases. (**D**) *P < 0.05; **P < 0.01; ^§^*P* < 0.001. In contrast, resPrP^D^ (**B** and **E**) generated very similar sedimentation equilibrium profiles in both sets of sCJDMM2- or sFI-inoculated TgGlyc− mice (**B**) mimicking the profiles of the original diseases (**E**). (**C**) TotPrP^D^ in TgGlyc+ mice replicated the original profile of sFI (**D**) following the inoculation of each of the two diseases (the amount of resPrP^D^ was insufficient for sedimentation equilibrium analysis). (**D** and **E**) Edited graphs published in Cracco *et al*.^19^**A**–**C**: *n* = 3 per group. Data are expressed as mean ± standard deviation. PrP^D^ = disease-related prion protein; Tg = transgenic.

Sedimentation equilibrium that assesses the density of the PrP aggregates showed that the gradient distributions profiles of totPrP^D^ (resPrP^D^+ PK-sensitive PrP^D^) and those of resPrP^D^ preparations generated in CJDMM2- and sFI-inoculated TgGlyc− mice were similar, with over 70% of the aggregates populating the high density (16–21) fractions (Fig. 6A and B). Both profiles mimicked the totPrP^D^ and resPrP^D^ distributions observed in the human sCJDMM2 (Fig. 6D and E). In contrast, the totPrP^D^ aggregate distribution of the sFI TgGlyc− recipients (but not the resPrP^D^ distribution) differed from that of the human disease where the lower density and PK-sensitive aggregates (fractions 4–8) accounted for >70% of the total (Fig. 6D and E). This finding suggests that the TgGlyc− mice totPrP^D^ cannot efficiently replicate the sFI low-density aggregates. Remarkably, the low-density totPrP^D^ aggregates were well represented in the TgGlyc+ mice exposed to either sCJDMM2 or sFI-generating profiles that were virtually identical to the original sFI profile in both conditions (Fig. 6C). The amount of resPrP^D^ recovered from the gradient experiments of TgGlyc+ mice was insufficient for the sedimentation equilibrium analysis.

#### Conformational solubility and stability assay

**Figure 7:**
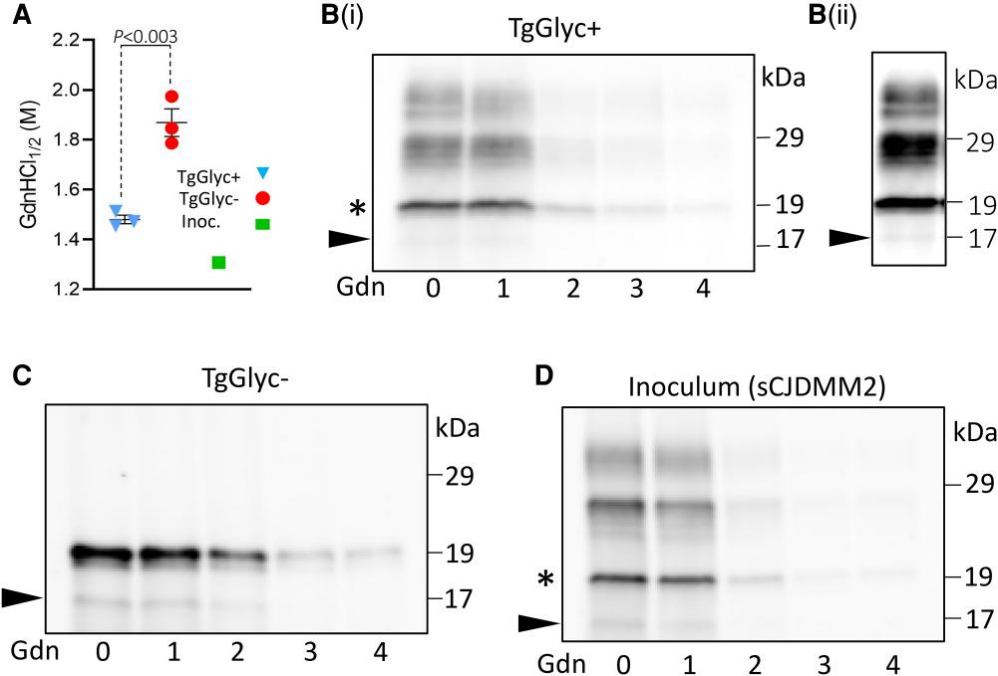
**Modified conformational solubility and stability assay of resPrP^D^ from TgGlyc+, TgGlyc− mice and sCJDMM2 inoculum.** In this version of the original conformational stability and solubility assay (CSSA),^19,35,37^ the concentration (in molar values) of guanidine-hydrochloride (GdnHCl) required to denature and render proteinase K (PK)-sensitive 50% of the initial resPrP^D^ is used as the resPrP^D^ stability index and referred to as [Gdn-HCl_1/2_]. (**A**) The stability index of resPrP^D^ recovered from the TgGlyc− mice (first passage) was significantly higher than that of the TgGlyc+ mice (1.87 ± 0.06 versus 1.48 ± 0.02, *P* < 0.003), while the resPrP^D^ stability index of the inoculum (Inoc.) was 1.31. [**B**(**i**)–**D**] The western blots confirm that the CSSA of the unglycosylated resPrP^D^ self-templating in the TgGlyc− mice differs in stability from the TgGlyc+ mice unglycosylated isoform (asterisk) despite their electrophoretic co-mobility (see also Fig. 5). (**D**) The stability index of the resPrP^D^ harvested from the sCJDMM2 inoculum mimicked that of the Glyc+ host. A faint band of 17 kDa, which appears to be slightly more intense in TgGlyc− preparations, likely represents the known anchorless resPrP^D^ isoform^36^ (arrowhead) [**B**(**ii**) longer exposure of lane 1 from **B**(**i**)]. Densitometry of the 17 and 19 kDa western blot bands, the only two bands detected, in TgGlyc− mice showed that the 17 kDa band accounted for 21.1% ± 5.7 (mean ± standard error) of the total. Tohoku-2 to human PrP residues 97–103 was used in the western blots. resPrP^D^ = resistant disease-related prion protein; Tg = transgenic.

The modified CSSA, which measures PrP^D^ stability as a function of the resistance to denaturants, demonstrated a significantly higher stability index of the sCJDMM2 resPrP^D^ converted in TgGlyc− mice at first passage compared to that of the glycosylated resPrP^D^ from the control mice (1.87 ± 0.055 versus 1.48 ± 0.017; *P* < 0.003). The stability index of the inoculum (1.31) was congruent with that of the control (Fig. 7A). The western blot study [Fig. 7B(i)–D], while providing visual support to the resPrP^D^ stability data, also revealed that in the TgGlyc+ mice and in the inoculum, the unglycosylated isoforms share the stability characteristics with the other two glycoform bands (di- and monoglycosylated), and that the stability of all three glycoforms similarly differ from the stability of the resPrP^D^ replicated in the TgGlyc−. Moreover, the western blot revealed the presence of a faint 17 kDa band that was slightly more noticeable in Glyc− mice preparation but present in all western blot preparations. Densitometry of the 17 and 19 kDa western blot bands in TgGlyc− mice, the only two bands detected in these mice, showed that the 17 kDa band accounted for 21.1%±5.7 of the total resPrP^D^ detected (Fig. 7).

All findings are summarized in Supplementary Table 1.

## Discussion

Sigurdson and colleagues used knock-in technology to engineer transgenic mice that expressed glycan-free mouse PrP at normal level by substituting N to Q at codons 180 and 196.^3^ These mice and wild-type controls were challenged with four mouse-adapted PrP^D^ strains: three of which, RML, 22L and ME7, were free of fibrils (i.e. subfibrillar), highly neuroinvasive and in wild-type mice, are commonly associated with spongiform degeneration and punctate or synaptic PrP^D^ deposition lacking plaques. The fourth strain, mCWD, was fibrillar and typically associated with the formation of prominent PrP^D^ plaque-like structures.^3^ After inoculation to the PrP glycan-free mice, each of the three subfibrillar strains showed augmented spongiform degeneration accompanied by the increase in size and density of the PrP^D^ deposits, while ME7 also caused neuronal loss and astrogliosis in the hippocampus. Moreover, the cerebral cortex, and in particular the hippocampus, were the most impacted compared to the cerebellum and brainstem. Few variations between glycan-free and control mice histotypes were detected following the inoculation of the fibrillary strain.^3^ These findings, in conjunction with additional PrP^D^ structural studies, led the authors to conclude that for at least the ME7 strain, the absence of glycans favoured the PrP^D^ cleavage from the cell plasma membrane. This event was mediated by the metalloproteinase ADAM10 and would lead to the subsequent enrichment in heparan sulphate, two conditions that favour the formation of plaques and plaque-like deposits also by subfibrillar strains.^3,46^

Our histopathological findings show similarities with those of Sigurdson and colleagues.^3,17^ At the first passage of sCJDMM2 and sFI strains, both TgGlyc− mice and controls accurately replicated the type of spongiform degeneration and the pattern of PrP deposition distinctive of both human diseases. However, these changes were significantly more severe in the TgGlyc− mice, where at sCJDMM2 first passage the spongiform degeneration score in the cerebral neocortex exceeded by over four times that of the normally glycosylated controls. The spongiform degeneration was accompanied by more prominent astroglial and microglial reactions, focal neuronal loss and the presence of conspicuous PrP aggregates in the hippocampal region, that were impacted by an intense glial reaction. Similar features were also observed in TgGlyc− mice challenged with the FFI strain which, at variance with the previous two diseases, did not transmit to control mice even after strain adaptation acquired through a previous passage in TgGlyc− mice. Notably, neither TgGlyc− mice nor controls reproduced the focal thalamic atrophy typically associated with both sFI and FFI. However, a PET metabolic study has identified thalamic hypometabolism, consistent with neuronal impairment, as the first and presymptomatic lesion in FFI.^47^ Should this be the case, this initial and naturally occurring lesion is likely to be bypassed in the experimental model of disease transmission, where the disease is triggered by the intracerebral inoculation of the seed. Finally, the finding that spongiform degeneration displayed the typical features only in transmission experiments requiring relatively long incubation periods suggests that spongiform degeneration undergoes a time-dependent evolutionary process.

A major distinction between our findings and those reported by the Sigurdson group,^3^ besides the histotype severity, is the duration of the incubation periods. They observed minimal or no reduction of the incubation periods at the first passage between glycan-free mice and controls challenged with two of the four PrP^D^ strains, while with the other two strains, the incubation periods were actually increased. Furthermore, the common shortening of the incubation period between the first and second passage occurred significantly with only one strain. In contrast, the average incubation periods in our TgGlyc− mice were reduced by >50% at the first passage in both sCJDMM2 and sFI experiments compared to the controls. Moreover, they further decreased by ∼71% (sCJDMM2) and ∼56% (sFI) at the second passage. No adaptation was observed at the third passage in the sCJDMM2, and possibly in the sFI transmission, if the second passage with short incubation is considered. Incubation periods as short as 80 dpi that we observed at the second and third passages are rare, perhaps unprecedented in transmission of human prion isolates to humanized TgGlyc+ mice, even with higher PrP expression levels than ours.^48^ The high conversion efficiency of the glycan-free PrP substrate is corroborated by the finding that resPrP^D^ recovered from TgGlyc− mice at sCJDMM2 first passage exceeded that of the glycan+ control mice by almost 40-fold. Although a decrease in PrP^D^ clearance may also play a role, given the prominent PrP deposits in the hippocampus, the amount of resPrP^D^ recovered from the TgGlyc− mice is astonishing, especially when considering that the incubation periods for the TgGlyc− mice, as well as their PrP expression rate, were half those of the TgGlyc+ mouse controls.

The remarkable efficiency of the TgGlyc− PrP substrate to convert to highly self-templating PrP^D^ is consistent with the findings of our previous *in vitro* amplification study where PrP extracted from TgGlyc− mice brain was used as substrate to amplify PrP^D^ seeds from major sCJD subtypes.^15^ The amplification efficiency achieved with the sCJDMM2 seed was 3.4 × 10^4^ higher than that obtained using substrate from TgGlyc+ mice, which were similar to those used as controls in the current study but had twice the PrP expression level.^15^ Combined, our *in vivo* and *in vitro* data underline the exceptional efficiency of human glycan-free PrP^D^ to self-replicate when challenged with PrP^D^-129MM, type 2. It has been suggested that the absence of significant incubation shortening observed by Sevillano and colleagues^3^ is due to the transmission barrier generated by the double mutation required to knockout PrP glycosylation.^46^ Furthermore, the mouse-adapted PrP^D^ strains used by Sevillano and co-workers^3^ might have given an advantage to the control mice. If the PrP N181Q/N197Q double mutation generates a transmission barrier, then our study indicates that it must be modest as it did not conceal the high self-replication efficiency of the glycan-free PrP^D^. Moreover, in our previous *in vitro* amplification study, we also reported that by replacing as substrate Glyc− PrP with wild-type PrP^C^ that was only partially deglycosylated (not rendered glycan-free) by PNGase treatment, the amplification efficiency decreased by only one degree of magnitude (10^3^ versus 10^4^).^15^ This finding minimizes any potential negative impact of the double mutation as transmission barrier while underscoring the role of glycans in reducing the conversion efficiency. Finally, our finding that the FFI PrP^D^ strain generated in TgGlyc− mice failed to replicate when it was back-passaged to the TgGlyc+ mice, like the human FFI PrP^D^ at first passage, also supports the pivotal role of the glycans (Table 1). It indicates that the high conversion efficiency of the Glyc− PrP is lost when the Glyc− PrP^D^ seed is re-exposed to the Glyc+ PrP^C^ substrate.

The western blot examination showed that the 19 kDa major band of resPrP^D^ from the TgGlyc− mice matched that of the unglycosylated resPrP^D^ isoform extracted from controls and inocula. This indicates that TgGlyc− PrP^D^ also belongs to type 2 PrP^D^, which has several implications. First, a previous study has shown that the 19 kDa resPrP^D^ type 2 fragment, which is associated with sCJDMM2, sFI and FFI, is generated by a major cleavage at the N-terminus PrP residue S97 with preservation of the C-terminus, including the anchor.^23^ Therefore, the shared electrophoretic mobility of resPrP^D^ from TgGlyc− mice with controls and the human diseases implies that the double mutation needed to knockout glycan expression in TgGlyc− mice does not interfere with the accurate replication of the regional transition from α-helix to β-sheet structure of the wild-type resPrP^D^ type 2, although the CSSA-determined stability index was quite different. Second, despite sharing this important conformational feature, the TgGlyc− and the unglycosylated wild-type resPrP^D^ self-replicate and accumulate at quite different rates. This suggests that the supposed restraining impact of the glycan presence in wild-type resPrP^D^ applies to all the resPrP^D^ glycoforms regardless of the presence and degree of glycosylation. Finally, according to Sevillano *et al*.,^3^ at least in two of their strains, Glyc− PrP is preferentially cleaved at the cell surface by the metalloproteinase ADAM10, generating anchorless PrP^D^. This would then be efficiently converted to anchorless Glyc− resPrP^D^ promoting the formation of amyloid plaques.^3^ Most human prion diseases have small amounts of anchorless resPrP^D^.^40^ In sCJDMM2, the anchorless resPrP^D^ variant has a 17 kDa western blot mobility (as it shares the N-terminus with the anchored variant) and accounts for ∼17% of the total resPrP^D^.^36^ Our western blot showed a thin 17 kDa band in all our preparations (Fig. 7). In TgGlyc− mice, densitometric analyses showed that the 17 kDa anchorless component accounted on average for 21.1 ± 5.7% of the total resPrP^D^, which is similar to the 17% reported in sCJDMM2.^36^ This suggests that the double mutation and the lack of glycans did not significantly affect the representation the of anchorless resPrP^D^ in our TgGlyc− mice compared to the human disease (Fig. 7). However, the ∼40× increase of the total resPrP^D^ in TgGlyc− mice indicates that even maintaining the normal ratio, the ADAM10-mediated pathway of PrP processing may have generated sufficient anchorless PrP to trigger the formation of the large PrP^D^ deposits that we observed in TgGlyc− mice with long incubation disease.

It is noteworthy that about half of the TgGlyc+ mice inoculated with isolates from two cases of sCJDMM2 self-templated PrP^D^ type 1 while all TgGlyc− mice challenged with the same seed were invariably associated with PrP^D^ type 2. As for the TgGlyc+ mice, this finding is not surprising as ∼50% of sCJDMM2 cases also display resPrP^D^ type 1, even if cases containing minute amounts of this resPrP^D^ type are included.^49^ As type 1 replicates faster, it may become the dominant or even the only detectable type upon transmission. Furthermore, the absence of resPrP^D^ type 1 in TgGlyc− following the challenge with the same inocula suggests that TgGlyc− PrP preferentially converts to PrP^D^ type 2 over type 1.

Other defining features of the glycan− PrP^D^ have emerged from the two sets of conformational studies. The comparative analyses of the sedimentation equilibrium profiles related to totPrP^D^ from the sCJDMM2 and sFI donors with those of the TgGlyc− and TgGlyc+ hosts suggest that both hosts lose the competence to replicate the distinct representations of low and high density aggregates that distinguish sCJDMM2 from sFI. The TgGlyc− mice seem to have limited capability to generate the low density aggregates that is a molecular signature sFI, favouring instead the formation of a high density aggregate population following exposure to both conditions. On the contrary, TgGlyc+ mice maintain the propensity to form low density aggregates, which were highly represented following inoculation of each of the two conditions.

Even more puzzling was the detection with CSSA of a significant increase in stability of the glycan− resPrP^D^ when compared with the control mice whose values were similar to those of the sCJDMM2 inoculum.^19^ However, the high degree of stability of resPrP^D^, also noted in one strain by Sevillano and colleagues,^3^ seemed to correlate with (i) the TgGlyc− mice histotype that is characterized by focal but relatively large deposition of plaque-like PrP^D^ at passages with long incubation periods; as well as (ii) the preference of the glycan− PrP^D^ to form high density aggregates as demonstrated with sedimentation equilibrium. However, a highly stable PrP^D^ strain counters the overall marked shortening of the incubation periods associated with the glycan− PrP^D^. Although the discrepancy between strain stability and disease duration is well recognized, this issue remains unresolved.^50^

Finally, the present findings also suggest that there is a correlation between the length of the incubation periods and the severity of the traditional histopathology, where in short incubation passages, the severity of spongiform degeneration and/or its typical features, as well as the PrP^D^ deposition, are reduced. This perplexing observation raises the issue of the toxic form of PrP^D^. In light of the recently reviewed mechanisms of prion toxicity pioneered by Collinge and colleagues, our findings are consistent with the postulated uncoupling of prion propagation, during which classic histopathology becomes prominent and the PrP toxic isoform, likely to be mono- or oligomeric, causes more subtle but deadly cellular lesions.^51-55^ If these observations are accurate, the TgGlyc− mouse could serve as a model to further test the prion toxicity hypothesis and to gain insights into the structural characteristics that distinguish the PrP^D^ controlling the histotype from the PrP^D^ toxic variant.

## Conclusions

The transmission of three related but distinct human diseases, sCJDMM2, sFI and FFI, to transgenic mice expressing human glycan− PrP has led to several conclusions:

- The TgGlyc− mouse that carries the N181Q/N197Q double mutation at the glycosylation sites is a valid model as we did not observe negative effects traceable to the point mutations nor any prion-unrelated impact due to the exclusive presence of unglycosylated PrP. Additionally, the TgGlyc− mouse model is more permissive as, unlike humanized control mice, it efficiently transmits the mutated FFI strain, although it failed to overcome the hamster–human species barrier.
- Even considering the possibility of a decrease in clearance, the glycan− PrP^D^ has shown a very high self-replication efficiency that exceeds control values by 40× at first passage, despite the ∼50% reduction in the incubation periods. Furthermore, the glycan− PrP^D^ is highly histopathogenic, resulting in a 4× more severe spongiform degeneration, accompanied by a severe astroglial and microglial response, and the propensity to produce large PrP^D^ aggregates.
- Glycan− PrP^D^ accurately reproduces the typical spongiform degeneration of the original human diseases, including its PrP immunostaining pattern, complemented by an apparently accurate replication of the resPrP^D^ type 2 secondary structure.
- Although the length of the wild-type resPrP^D^ fragment and the presence of the 17 kDa anchorless variant associated with the original disease are replicated, the glycan− PrP^D^ displays distinct conformational features, including high stability and the tendency to form sedimentation equilibrium high density aggregates, further supporting the conformational distinction between Glyc− and Glyc+ PrP^D^.

These findings confirm the protective impact of the PrP^C^ glycans, that make PrP^D^ less infective and destructive while playing a lesser role in the histotype determination. However, issues that need clarification include the apparent contradiction between the very efficient transmission and the high stability of the glycan− PrP^D^ human prion strain as well as the seemingly paradoxical decrease in severity of the traditional histopathology at passages with very short incubation periods pointing to the presence of a toxic conformer. Future studies will clarify whether the glycan− PrP^D^ features that we observed also apply to other human prion diseases or are type and subtype dependent.

## Supplementary Material

awad399_Supplementary_Data

## Data Availability

The data that support the findings of this study are available from the corresponding authors (L.C. and P.G.), upon reasonable request.
