# Supplementary material for: Efficient transmission of human prion diseases to a glycan-free prion protein-expressing host
Source: Brain. 2023 Nov 24;147(4):1539–52. doi: 10.1093/brain/awad399 (PMC10994570; doi:10.1093/brain/awad399)

Efficient transmission of human prion diseases to a glycan-free prion protein-expressing host

Laura Cracco,[1] Ignazio Cali,[2,3] Mark L. Cohen,[2] Rabail Aslam,[2] Silvio Notari,[2] Qingzhong Kong,[2,3,4] Kathy L. Newell,[1] Bernardino Ghetti,[1] Brian S. Appleby,[2,3,4,5] Pierluigi Gambetti[2]

[1] Department of Pathology and Laboratory Medicine, Indiana University School of Medicine, Indianapolis, IN 46202, USA.

[2] Department of Pathology, Case Western Reserve University, School of Medicine, Cleveland, OH 44106, USA.

[3] National Prion Disease Pathology Surveillance Center, Case Western Reserve University, School of Medicine, Cleveland, OH 44106, USA.

[4] Department of Neurology, Case Western Reserve University, School of Medicine, Cleveland, OH 44106, USA.

[5] Department of Psychiatry, Case Western Reserve University, Cleveland, OH 44106, USA.

Address correspondence to:

Pierluigi Gambetti: Department of Pathology, Case Western Reserve University, School of Medicine, Cleveland, OH 44106, USA. Phone 216.410.6883; Email: pxg13@case.edu

Laura Cracco: Department of Pathology and Laboratory Medicine, Indiana University School of Medicine, Indianapolis, IN, 46202, USA. Phone: 317.274.1658; Email: lcracco@iu.edu

Supplementary Table 1: Summary of the main findings

Parameters	Sporadic CJD MM2	Sporadic Fatal Insomnia	Familial Fatal Insomnia
**Histopathology in human diseases**	SD: Large, confluent vacuoles w. PrP deposits, preferentially in the neocortex	SD: Similar to sCJDMM2 but less severe, its presence depends on disease duration (>24 months). Focal thalamic atrophy	SD: Similar to sCJDMM2 but much less severe and strictly dependent on disease duration. Focal thalamic atrophy
**Inoculated mice**			
**Glyc- mice average incubation periods (in dpi)**	1stp.: 266 (~2x shorter than Glyc+ controls). 2nd & 3rd p.: 78	1stp.: 346 (~1.8x shorter than Glyc+ controls. 2nd & 3rd p.: 114	1st p.: 289 (no transmission in controls). 2nd p.: 193
**Histopathology in Gyc- mice and in Glyc+ controls**	SD & PrP deposition similar to hu. disease; ~4x more severe than controls, w. large PrP deposit & high astro-microglia reactions	Same as after sCJDMM2 transmission and more severe than in controls	Same as in sFI-Glyc- mice but slightly less severe
**Histopathology severity & incubation period**	Severity, accurate SD replication and presence of the large PrP deposits directly correlated w. the length of the incubation period		
**resPrP^{D} WB in Glyc- mice**	Same electrophoretic mobility as unglycosylated in Glyc+ and 3 hu. diseases		
**resPrP^{D} quantitative assessment in Glyc- mice**	~40x > Glyc+ control	Definitely > controls	High; no controls avail.
**Tot & resPrP^{D} SE profiles**	Glyc- mice: prevalence of high-density aggregates Glyc+ mice: prevalence of low-density aggregates		Not tested
**Glyc- mice: CSSA**	Significant increase in resPrP^{D} stability	Not tested	Not tested
**Glyc- mice: Anchorless resPrP^{D} as % of total**	21.1: Similar to that of the sCJDMM2 (17.4)	Not tested	Not tested

Abbreviations: avail.: available; CSSA: conformational solubility and stability assay; dpi: days post-inoculations; Glyc-: TgGlyc mice; hu.: human; mo.: months; p.: passage; SD: spongiform degeneration; w.: with; WB: western blot; SE: sedimentation equilibrium.

Supplementary Figure 1 Original uncropped immunoblots shown in Figures 5 A, and 5 B. Red boxes indicate the cropped region shown in the corresponding panel.

Fig. 5 A

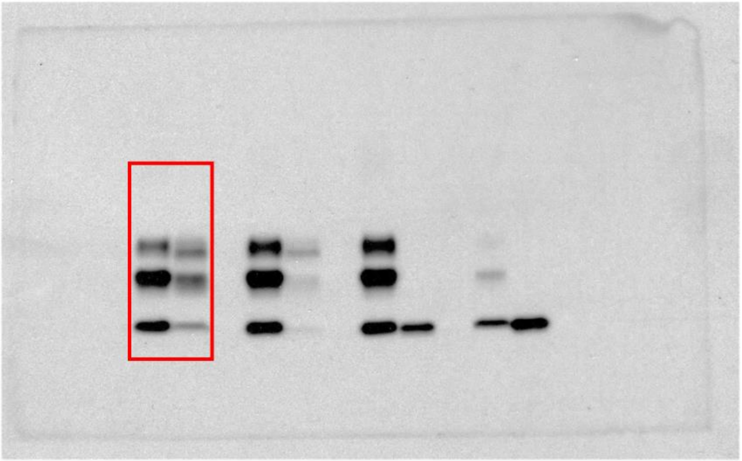

Panel 1

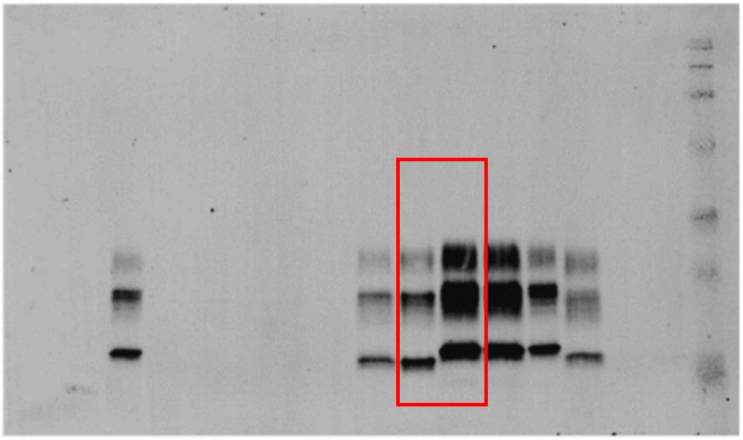

Panel 2

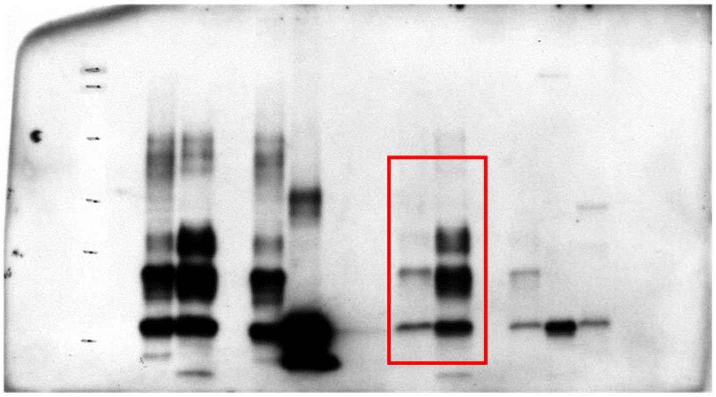

Panel 3

Fig. 5 B

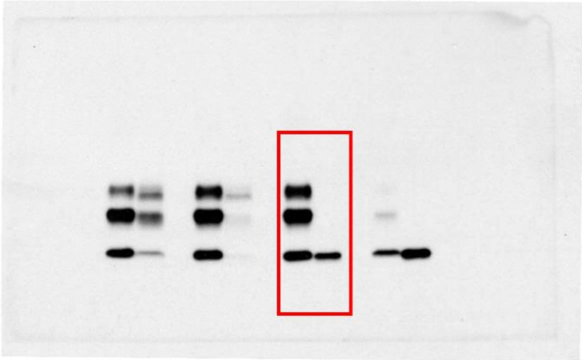

Panel 1

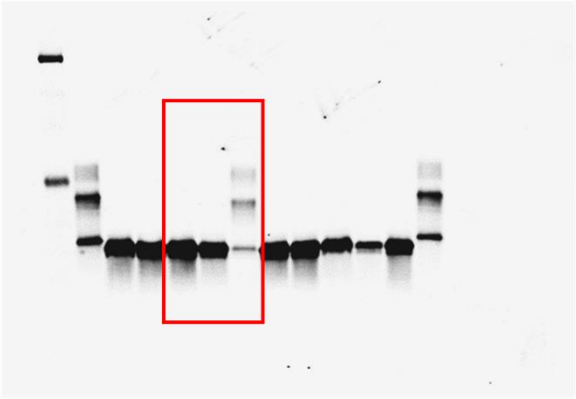

Panel 2

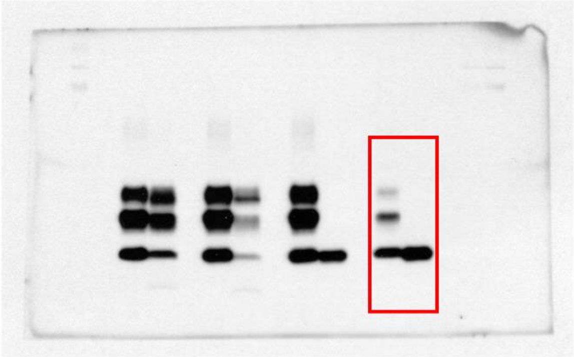

Panel 3

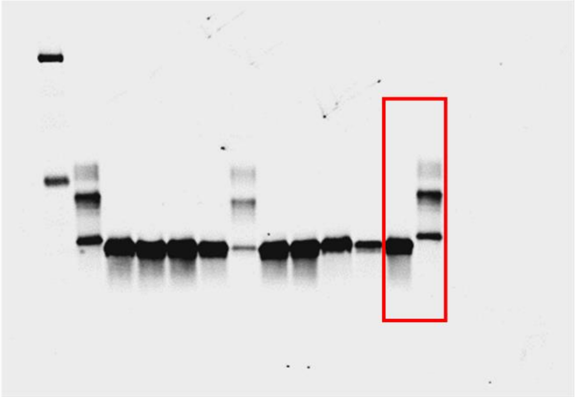

Panel 4

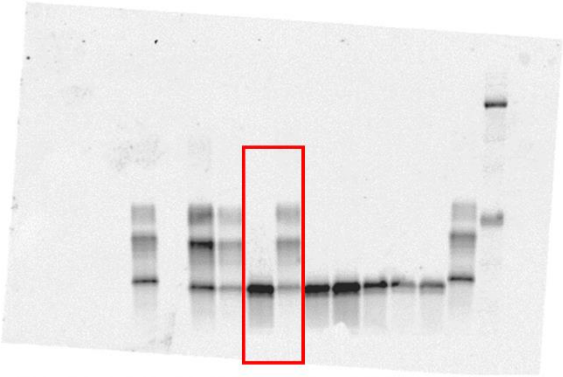

Panel 5

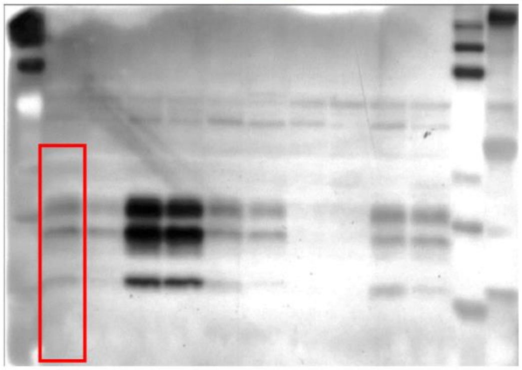

Panel 6

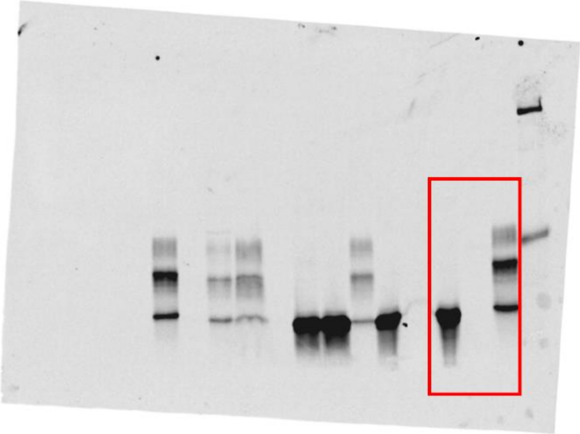

Panel 7

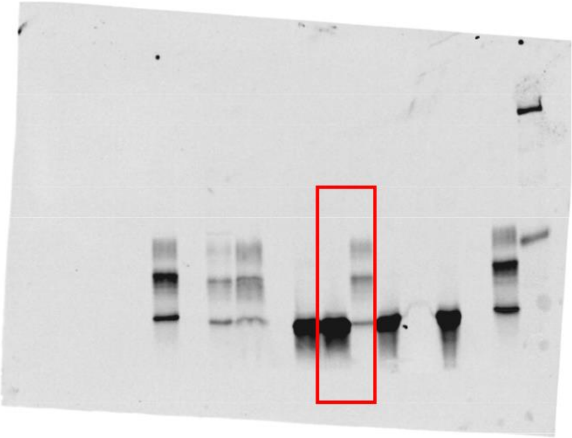

Panel 8

Supplementary Figure 2 Original uncropped immunoblots shown in Figures 7 B(i), 7 B(ii), 7 C and 7 D. Red boxes indicate the cropped region shown in the corresponding panel.

Fig. 7 B(i)

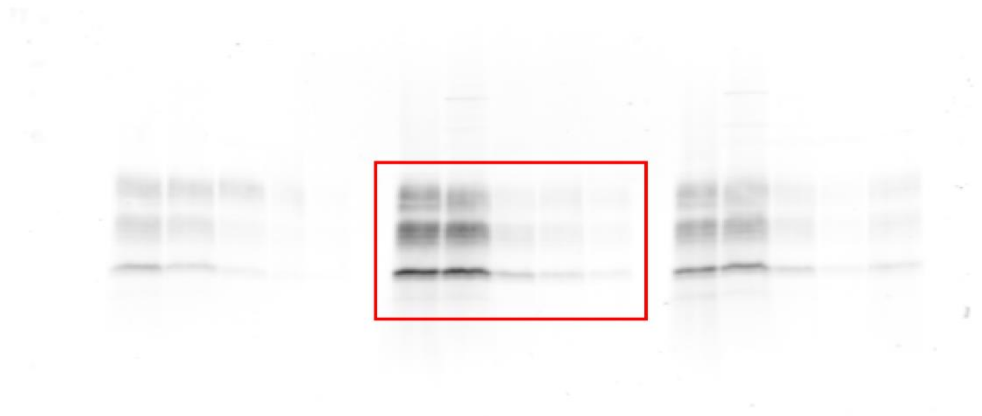

B(i)

Fig. 7 B(ii)

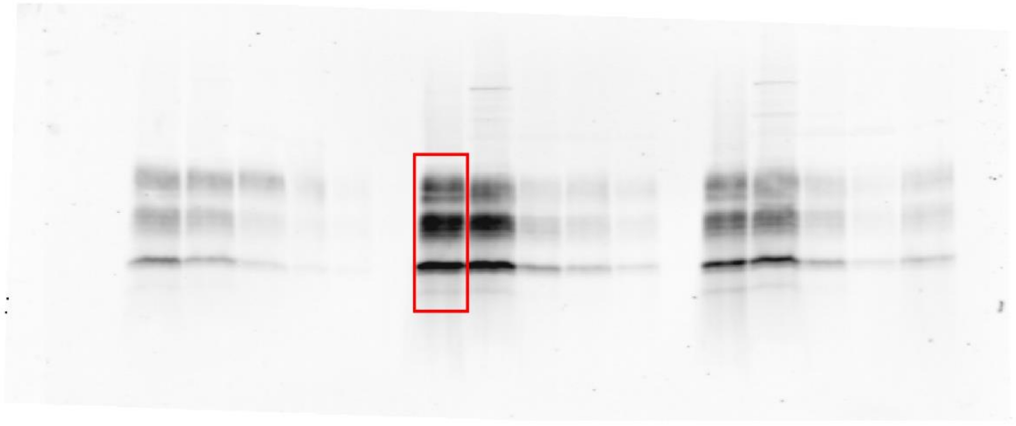

B(ii)

Fig. 7 C

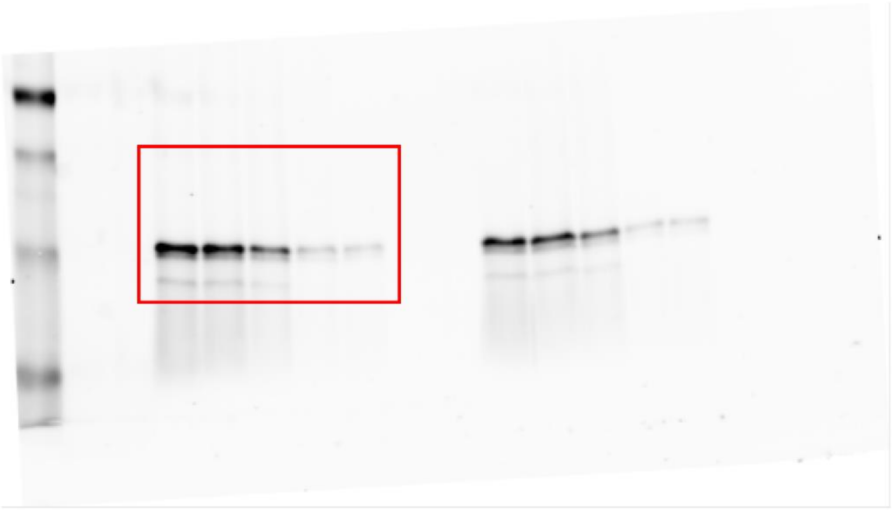

Fig. 7 D

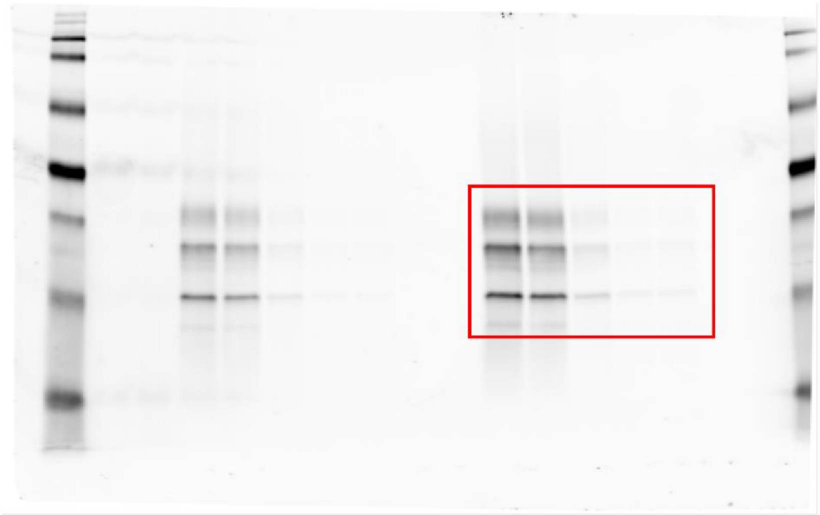

Supplement: awad399_Supplementary_Data [file awad399_supplementary_data.pdf]
